# Echocardiography in the Assessment of Heart Failure Patients

**DOI:** 10.3390/diagnostics14232730

**Published:** 2024-12-04

**Authors:** Frank L. Dini, Matteo Cameli, Andrea Stefanini, Hatem Soliman Aboumarie, Matteo Lisi, Per Lindqvist, Michael Y. Henein

**Affiliations:** 1 Istituto Auxologico IRCCS, 20149 Milan, Italy; frank.dini@santagostino.it; 2 Division of Cardiology, Department of Medical Biotechnologies, University of Siena, 53100 Siena, Italy; matteo.cameli@unisi.it (M.C.); astefanini94@gmail.com (A.S.); 3 Department of Anaesthetics, Critical Care and Mechanical Circulatory Support, Harefield Hospital, Royal Brompton and Harefield Hospitals, London UB9 6JH, UK; hatem.soliman@gmail.com; 4 School of Cardiovascular, Metabolic Sciences and Medicine, King’s College London, London SE1 9RT, UK; 5 Division of Cardiology, Department of Cardiovascular Disease-AUSL Romagna, Ospedale S. Maria delle Croci, 48121 Ravenna, Italy; matteo.lisi@auslromagna.it; 6 Department of Diagnostics and Intervention, Clinical Physiology, Umea University, 90585 Umea, Sweden; per.lindqvist@umu.se; 7 Imperial College London, Du Cane Road, London W12 0NN, UK

**Keywords:** heart failure, left atrial function, cardiac output, left ventricular filling pressure, speckle tracking echocardiograph

## Abstract

Doppler echocardiography is the corner-stone of non-invasive investigation of patients with a clinical diagnosis of heart failure. It provides an accurate and quantitative assessment of cardiac structure and function. Furthermore, spectral Doppler measurement is an invaluable technique for estimating intracardiac pressures with their crucial value in the optimum management of heart failure patients, irrespective of ejection fraction. Speckle tracking echocardiography stretches the unique application of echocardiography to analyze the myocardial deformation function which has proved very accurate in detecting ischemia, dyssynchrony, subclinical dysfunction and also in estimating pulmonary capillary wedge pressures. The role of longitudinal myocardial left atrial deformation dynamics has recently emerged as a valuable tool for assessing left ventricular diastolic dysfunction in patients with cardiac diseases regardless of their ejection fraction. Finally, the extent of myocardial deformation has been shown to correlate with the severity of myocardial fibrosis, a common finding in patients with heart failure.

## 1. Left Ventricular Function in Heart Failure

Heart failure (HF) is a chronic progressive disease that concerns over 64 million people, and on current estimates, based on hospital or community records, places prevalence at between 1% and 3% of the population [1,2]. The basis of the failing heart is the inability to deliver oxygen and nutrients to the tissues to support their metabolic needs or to provide forward flow with an excessive increase in filling pressures. Although evidence-based therapies have significantly improved, the rate of mortality remains high. It is therefore important to characterize HF patients using the widely available diagnostic tools, such as Doppler echocardiography, to fulfill the patients’ unmet clinical needs in tailoring medical treatment and optimizing the indications for interventions.

Nowadays, HF can be phenotyped by echocardiography and this may be useful for prognostication as well as guiding treatment [3]. Echocardiography represents a unique means for investigating both systolic or diastolic function; the most important echocardiographic methods with their advantages and disadvantages are summarized in Table 1. Patients with HF are classified according to ejection fraction (EF) as follows: reduced EF (HFrEF), where EF is ≤40%, mildly reduced EF (HFmrEF), where EF ranges between 41% and 49%, and preserved EF (HFpEF) where EF is ≥50%, and with improved (HFimpEF) where EF was <40% at first assessment then increased by ≥10 points to >40% at after treatment [4]. Diastolic dysfunction may also be present with varying degrees in all subtypes of HF [5], from patients with HFrEF to those with HFpEF, the latter being more prevalent with advancing age and particularly in females [6].

## 2. Assessing Systolic Function

The contracting left ventricle (LV) pumps the blood into the systemic circulation to a high peak outflow velocity. The volume of blood ejected from the ventricle with each beat (the stroke volume: SV) results from the extent of myocardial fiber shortening, while cardiac power is related to both the SV and the pressure that is generated. The ventricle does not empty completely, and the volume remaining in end-systole, after the SV is expelled, is called the end-systolic volume (ESV). The SV indicates the amount of the initial volume ejected in systole. The LV EF is estimated from the end-diastolic volume (EDV) and ESV by applying the following equation:

$$EF=\frac{EDV-ESV}{EDV}\times 100$$

EF is the most widely used index for evaluating LV systolic performance. The normal values in adults, at rest, is between 50% and 70%. Its simplicity and good correlation with the patient’s outcome have made it a convenient tool in daily clinical practice. However, LV EF also has relevant limitations, including load dependency and the lack of any relationship with patients’ signs and symptoms. Moreover, LV EF estimation suffers from modest reproducibility since its determination is dependent on geometrical assumptions and on endocardial border detection [7].

Cardiac output (CO) is an extremely important cardiovascular measure that is closely related to the metabolic rate. For most healthy adults, the resting heart rate is between 50 and 90 beats, while CO at rest is generally 5–7 L per minute. CO is determined by the SV and the heart rate:

*Cardiac Output* = *Stroke Volume* × *Heart Rate*

Doppler echocardiography is an ideal non-invasive tool for the quantitative assessment of SV and related parameters, including CO and cardiac index. Two dimensional echocardiography can be used to measure ESV and EDV and then SV can be calculated. Alternatively, an estimation of SV can be obtained by Doppler echocardiography [8] as the product of LV outflow tract (LVOT) flow velocity and its cross-sectional area (CSA). As the flow velocity varies through ejection, it is estimated by its velocity time integral (VTI). The latter measure is easily obtained by the echo machine from the area within the spectral Doppler curve. Once LVOT diameter (D) is determined and the LVOT VTI is calculated, the SV can be estimated by multiplying the LVOT-CSA by the VTI:

*Stroke volume* = *CSA* × *VTI* = *π r*^2^ × *VTI* = 0.785 × *D*^2^ × *VTI*

The quantification of SV by the continuity equation has the greatest potential of error since it depends on LVOT diameter assessment, which must be squared to obtain CSA. To limit technical shortcomings, some authors proposed a LVOT VTI < 15 cm as a cut off value for reduced SV [9]. The SV index (SVI) is another parameter that can be used to assess LV systolic performance. In patients with HF either in the acute or in the chronic setting, the risk of events significantly increases with SVI < 30 mL/m^2^, with a further rise in risk with SVI < 25 mL/m^2^, thus identifying a subgroup of HF patients with a severely compromised LV systolic function [10].

Myocardial strain (S) is a speckle tracking-based dimensionless index of deformation describing the percentage change in length (l) of a myocardial segment relative to its baseline length (l_0_):

$$Ε=\frac{\left(l-l_{0}\right)}{l_{0}}=\frac{\Delta l}{l_{0}}$$

Myocardial strain rate is the rate at which myocardial deformation takes place and is expressed as 1/s, but is rarely used in clinics.

Global longitudinal strain (GLS) measured by speckle-tracking echocardiography (STE) has been shown to be a more sensitive marker of LV systolic function than LV EF, with a higher predictive value for clinical outcome in optimally treated patients with HF and a broad range of LV EF [11,12]. One of the reasons why a reduction in LV GLS often precedes a decrease in EF is that myofibers that account for longitudinal shortening are located mainly in the vulnerable sub-endocardium. In addition, patients with increased LV mass frequently exhibit a reduction in GLS even when EF is still within the normal range [13].

The combination of LV GLS with the echo-hemodynamic profiles based on measures of SVI, LV filling pressures and right ventricular (RV) function further improves the prognostic stratification of HF patients with reduced LV EF (HFrEF), especially in those who are hemodynamically stable [14]. Moreover, the evaluation of LV GLS plays an important role in the detection of subclinical alterations in myocardial function [15].

## 3. Myocardial Energetics

Energetics is another integral component for characterizing myocardial function. Myocardial work is a pillar measure of myocardial function. In assessing energetic LV function, two types of work must be considered: the external work and the internal work. The total external work of the LV depends on the amount of pressure (pressure–volume [PV]-work) and the kinetic energy it generates. The ejection of a volume of blood under pressure represents the PV work; this is because, in a fluid system, work (force × distance) is equal to pressure × volume. It corresponds to the stroke work (SW), i.e., the PV work exerted by the LV per beat, that can be calculated as the SV times the blood pressure (BP) at which blood is ejected: The evaluation of the interaction between the LV and the arterial system can be expressed by a PV loop, which plots the changes in LV pressure and volume occurring during a single cardiac cycle [16].

**Figure 1:**
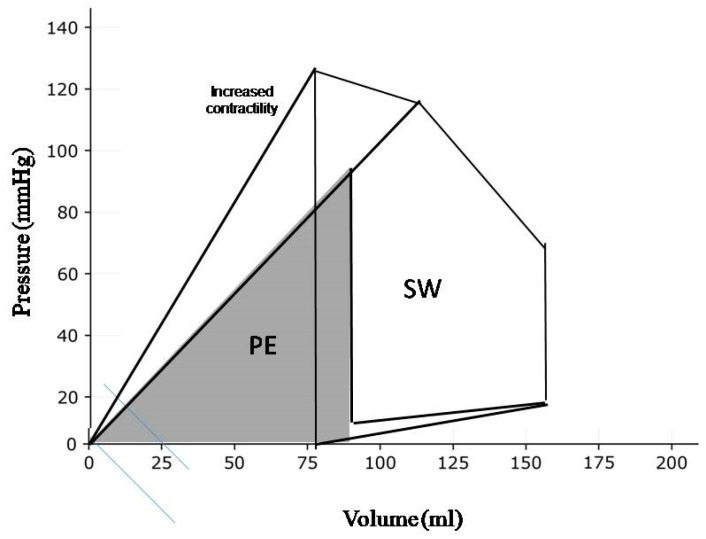
The components of left ventricular work. The stroke work (SW) corresponds to the energy that propels blood from the left ventricle into the aorta. The potential energy (PE) is the energy that is not converted in stroke work. Pressure–volume area (PVA) is the sum of stroke work and potential energy.

SW is reflected by the area of the PV loop and is proportional to both the pressure generated by LV contraction and the SV (Figure 1). The same SW can be performed by various loading conditions giving combinations of SV and intraventricular pressure. Changes in LV contractility can be appreciated by the counterclockwise rotation of the slope of the end-systolic PV relation (ESPVR). The slope of the ESPVR defines the LV end-systolic elastance. The crossing point where the slope of the ESPVR intersects with arterial elastance defines the ventricular–arterial coupling [17].

The goal of the cardiac metabolism is to produce chemical energy to supply the heart function. As with any mechanical pump, only a fraction of this chemical energy is transformed into external work, i.e., SW. The ventricle uses a large amount of energy to perform internal work. It includes energy used to stretch elastic elements and to overcome the inertia of the viscous components of the myocardium. This energy does not contribute to the propulsion of blood and it is finally degraded into heat. The internal work corresponds to the potential energy (PE), that can be graphically expressed by the PV triangle obtained by joining the end-systolic PV point to the origin. The total mechanical energy generated by ventricular contraction is the sum of SW and PE: (global myocardial work—GMW):

*Global Mechanical Work* = *SW* + *PE*

The area subtended by both internal and external work, i.e., GMW, correlates closely with myocardial oxygen consumption (MVO_2_) [18].

## 4. Echocardiographic Evaluation of Myocardial Energetics

Although an accurate assessment of cardiac energetics by PV analysis requires invasive measurements combined with loading interventions, the importance of this topic makes its simplified quantification by echocardiography potentially very useful.

Non-invasive methods for estimating PV relationships, including echocardiography, have been developed. The advantages of a non-invasive methodology are apparent [19]. It can be applied to large numbers of patients and assessment can be performed to evaluate changes in cardiac function chronically and serially, observing the natural history of the disease or the response to pharmacological or device-based therapy.

Pressure–strain loop (PSL) analysis is a novel noninvasive method for evaluating MW that combines information derived from strain imaging, assessed by speckle tracking echocardiography (STE) and systolic/diastolic blood pressure. It requires the assessment of the valvular opening and closure events that allow the determination of the isovolumic relaxation time and the ejection time. GMW is characterized by two distinct components: the global wasted work (GWW), that represents LV work that does not contribute to LV ejection, i.e., the internal work; and the global constructive work (GCW), that corresponds to the SW, that quantifies the energy consumed by the myocardium that effectively contributes to CO [20].

Mechano-energetic efficiency may be defined as the ratio of the external work accomplished to the consumed energy. The mechano-energetic myocardial efficiency (MME) of the LV to pump blood to supply tissues’ needs ranges between 14% and 35% at resting outputs and it is similar in all mammals [21]. Therefore, the amount of mechanical work, i.e., SW, provided by any mammal correlates with its metabolic rate. It is apparent, though, that MME is greater when the SV is high and the heart rate is low, while enlarged ventricles are generally characterized by a lower MME because of the elevated energy cost of LV performance due to increased internal work and MVO_2_.

Several methods have been proposed to assess MME, but many of them are difficult to acquire or do not accurately reflect the components of mechanical efficiency, especially myocardial oxygen uptake.

**Figure 2:**
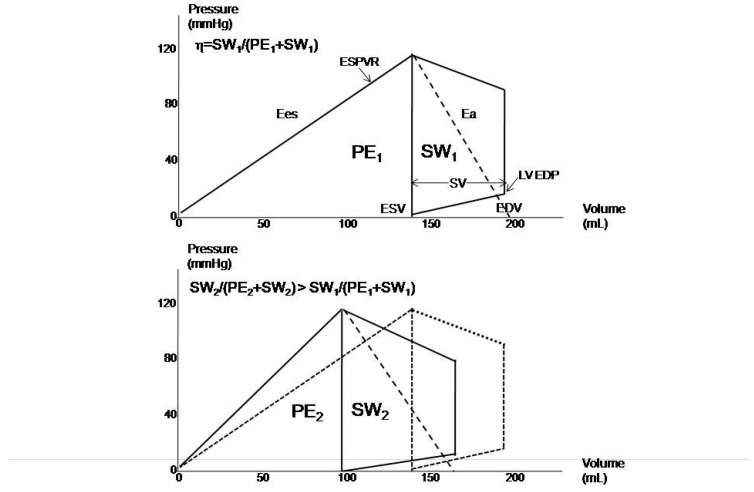
Improvement in myocardial mechanical efficiency (η) in patients (n = 150) exhibiting left ventricular reverse remodeling after six months of therapy with sacubitril/valsartan. Ea: arterial elastance; Ees: end-systolic elastance; EDP: end-diastolic pressure; EDV: end-diastolic volume; ESPVR: end-systolic pressure–volume relationship; ESV: end-systolic pressure; FU: follow-up; PE: potential energy, SW: stroke work; VA: ventricular–arterial.

**Figure 3:**
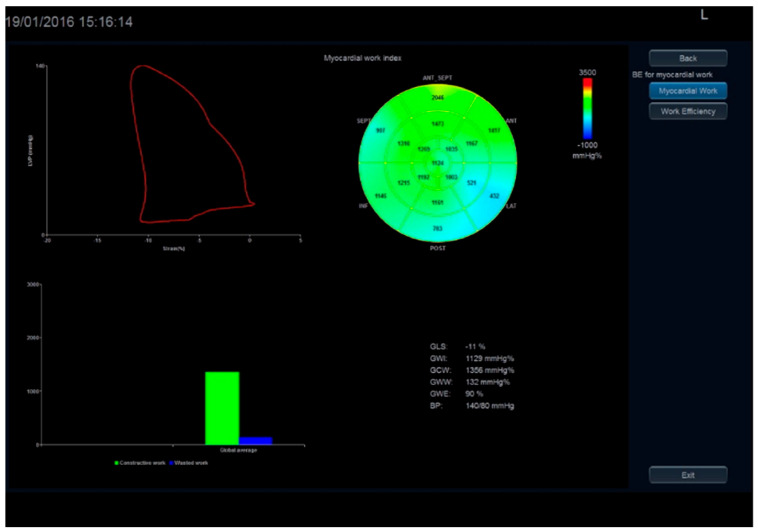
Myocardial work assessed by 2D strain.

From the echocardiography-derived PV loops, MME can be described as the ratio between SW and the sum of PE and SW (Figure 2). This analysis appreciates the positive effects of LV reverse remodeling, including the benefit to survival, exerted either by drug or device therapies, mediated by the improvement in MEE, which must be considered a common denominator of the beneficial effects [22]. As is apparent from Figure 3, reverse remodeling not only improves MME but also exerts a favorable impact on ventricular–arterial coupling. Additionally, a close relationship between MME and ventricular–arterial coupling in patients with HFrEF was recently observed to demonstrate the importance of a proper balance between LV performance and load in optimizing myocardial function.

Similarly, MME may be assessed by the PSL analysis of the quotient of the constructive work and the total (constructive and wasted) work (Figure 3). The formula for PSL-derived MME is GCW divided by the sum of GCW and GWW:

MME = GCW/(GCW + GWW)

**Figure 4:**
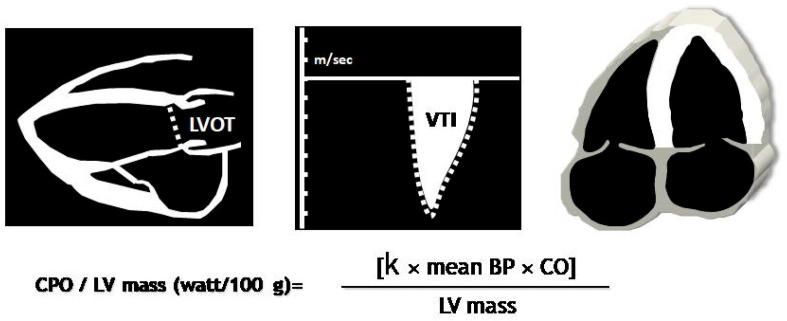
Cardiac power output normalized by LV mass can be determined using echocardiography as 0.222 X cardiac output X mean BP, where 0.222 is the conversion constant to W/100 g of LV myocardium.

Despite the feasibility of deriving PV loops from routine echocardiography, stress echocardiography may provide more information related to a comprehensive evaluation of the functional status of patients and to predict outcome. Cardiac power output (CPO) is a descriptor of cardiac function, derived from blood pressure and CO, that reflects the rate of SW performed by the left ventricle. LV pumping capability can be defined as the maximum level of CPO the ventricle can achieve, which can be non-invasively assessed during exercise or pharmacological stress echocardiography (Figure 4). Peak CPO-to-LV mass can be viewed as a surrogate marker of MME, since this ratio incorporates peak SW per unit time (i.e., CPO) and the maximal work (internal and external) that theoretically is inherent to 100 g of LV mass (W/100 g).

CPO-to-LV mass at peak stress may reflect the energy delivery of LV myocardium [23]. Peak CPO-to-LV mass will increase to a point, then decline as the power to mass becomes unfavorable. Patients whose increased LV mass is proportional to the peak CPO-to-LV mass should be identified as bearing a compensatory pattern of LV hypertrophy, whereas patients in whom their pumping capability does not parallel the extent of LV hypertrophy are likely to have inadequate, dysfunctional hypertrophy. The prognostic value of peak CPO-to-LV mass has been extensively validated in patients with cardiac disease either with reduced or preserved LV EF [24,25]. A peak CPO-to-LV mass ≤ 0.60 W/100 g predicted an unfavorable outcome in HF patients.

Finally, twisting and untwisting of the LV are concepts that became familiar to echocardiographers with the appearance of STE and boosted awareness of the fundamental features of myocardial mechanics. The LV twists in systole storing potential energy and untwist (recoil) in diastole releasing energy; as a result, twist aids LV ejection and untwist aids relaxation and cavity filling. Although the key role of LV twisting/untwisting in cardiac physiology is undisputable, its significance in daily clinical practice remains to be established. A decrease in LV twist has been reported in patients with cardiomyopathies in the presence of LV scar or dilation. It has been suggested that increased LV twisting in patients with hypertrophic cardiomyopathy may reflect the development of subendocardial ischemia. These findings highlight the importance of assessing LV twisting/untwisting in the diagnostic work-up of patients with cardiomyopathies [26].

## 5. Assessing Diastolic Function

The diastole is devoted to the pursuit of optimal ventricular filling through the process of LV myocyte relaxation and the additional contribution of the atrial systole. Myocyte LV relaxation and the left atrial (LA) contraction generate the LA–LV pressure differences that allow the blood to fill the ventricle during three phases; early diastole, diastasis and atrial systole.

The importance of studying diastolic function, dysfunction and failure makes echocardiography a very important tool with which to characterize and risk stratify HF patients. Diastolic dysfunction results from impaired LV relaxation, LV chamber stiffness, and ultimately increased filling pressure.

Although the uses of pressure tip manometers in the LV and/or the invasive assessment of pulmonary capillary wedge pressure (PCWP) have allowed us to measure the hemodynamic parameters, cardiac catheterization is an invasive procedure that occasionally results in serious complications. Doppler echocardiography can provide a safe alternative to invasive hemodynamic assessment. Much interest has been taken in the noninvasive estimation of left heart filling pressures: mean LA pressure (or PCWP) and LV end-diastolic pressure (LVEDP). Such an approach helps in assessing not only the intracardiac chamber pressure but also its compliance and myocardial function, thus presenting a comprehensive appraisal of cardiac physiology in a highly reproducible fashion.

The interpretation of Doppler echocardiographic information has increased our understanding of LV diastolic function [27]. The isovolumic relaxation time (IVRT) is the time interval between the closure of the aortic valve and the opening of the mitral valve, i.e., when the two valves are closed and there is no blood entering or exiting the LV; and can be easily estimated.

The impaired relaxation pattern is characterized by a mitral E/A ratio ≤ 0.75, a prolonged E wave deceleration time (EDT) and an IVRT usually > 100 ms. Relaxation abnormalities may impact LV filling in the elderly, in the presence of normal filling pressures, manifested as reduced E wave velocity, increased A wave velocity, E/A ≤ 0.75, and prolonged EDT. IVRT prolongs with age, as LV relaxation slows.

Dyssynchrony and incoordination can occur between myocardial relaxation, ventricular filling and atrial systole and between the different ventricular segments in the diastolic phases. Dyssynchrony may worsen in other conditions, e.g., hypertension and coronary artery disease, hence impacting LV filling pattern. These alterations largely depend on structural changes occurring at the myocardial level due to age and disease-related collagen deposition [28]. Other clinical conditions can also worsen these changes, including pressure afterload, i.e., hypertension and aortic stenosis as well as diabetes and coronary artery disease [29].

When diastolic dysfunction progresses, especially if it is associated with an accelerated heart rate, a summation filling pattern occurs and this may result in a reduction in SV. As a result, patients may became symptomatic and often do not adequately respond to beta blockers and heart rate slowing medications. With further deterioration of the passive myocardial properties, the LV chamber becomes stiffer, thus resulting in a raising of the filling pressures. This may be eventually followed by the backward transmission of the elevated pressure regimen to the pulmonary vascular bed with the occurrence of post-capillary pulmonary arterial hypertension, possibly followed by RV overload. Such changes may have a direct impact on the IVRT and on mitral inflow velocity patterns. In patients with an abnormal relaxation pattern, increased loading conditions—especially in the presence of decreased LV compliance—may be responsible for an elevation in the LA pressure necessary to maintain the early LA–LV driving pressure, and this is followed by changes in LV filling dynamics. This accounts for increased mitral E velocity, E/A ratio of 1 to 1.5 and normalized EDT (pseudonormal pattern). IVRT is generally shorter than normal (<80 ms). As diastolic dysfunction further progresses, there is a reduction in LV compliance and this results in early cessation of filling due to rapid equalization of LA and LV pressures. Hence, faster and more pronounced increases in LA pressure are necessary to maintain the driving pressure to a degree that allows LV filling to accommodate in a stiffer ventricle. The resulting mitral flow velocity profile—characterized by marked elevation of E velocity, shortened EDT (<140 ms) with an increased E/A ratio—is referred to as the restrictive pattern [30]. Patients with restrictive filling usually complain of dyspnea and some may manifest the consequences of reduced SV and low systemic blood pressure. The suppressed SV is not only caused by reduced LV systolic function but by the ventricular interaction and suppression of early diastolic RV filling which limits the SV going to the left heart [31].

Similar changes in LV function could result from electric disease manifested as prolonged depolarization (broad QRS), irrespective of the bundle branch block (BBB) pattern. Delayed and prolonged depolarization results in delayed segmental mechanical function, raised cavitary tension in early diastole and hence suppression of cavity filling in that phase. Such changes could be seen in patients with prior myocardial infarction [32] or dilated cardiomyopathy, hence the need for electric treatment and correction of the depolarization delay by optimum pacing. Patients with severe LV disease and broad QRS may also develop a prolonged PR interval. The combination of the two electric disturbances, broad QRS and prolonged PR interval, may result in long mitral regurgitation with pre-systolic component which shortens LV filling and reduces SV, particularly when the heart rate is fast. If these patients do not respond to heart rate-slowing medications, they should benefit from DDD pacing with short A-V delay.

Patients with HF and preserved ejection fraction (HFpEF) do not necessarily present with the same degree of diastolic disturbances as those with HF and reduced EF (HFrEF), since the former do not have very poor myocardial systolic function but the cavity tends to be stiff and filling pressures raised; hence, the more frequently seen atrial arrhythmia in these patients [33]. Likewise, dyssynchrony is not a common feature of HFpEF patients compared to HFrEF. These findings should guide towards the best treatment management once the nature and extent of the disturbed physiology are clearly identified and are able to explain patient’s symptoms.

## 6. Diastolic Function in Heart Failure

For a non-invasive estimation of filling pressures, a number of echocardiographic measures have been suggested in recent recommendations [34]. It is apparent that additional diagnostic information—beyond that contained in mitral flow velocity recordings—may be obtained from the study of pulmonary venous flow (PVF) velocities, tissue Doppler imaging (TDI), combined analysis of mitral and TDI and estimated pulmonary artery pressures, either by RV-right atrial retrograde pressure drop from tricuspid regurgitation or pulmonary-RV early or late diastolic pressure drop from continuous Doppler pulmonary regurgitation trace [35,36,37].

In patients with HFpEF, a pattern of abnormal relaxation can be associated with the elevation of LV filling pressures, especially in the presence of an enlarged LA (volume > 34 mL/m^2^), a ratio of E/averaged myocardial early velocity (averaged E/e′) > 14, an increased difference in the duration of PVF and the mitral flow velocity at atrial contraction (>30 ms), and elevated systolic and/or diastolic pulmonary pressures [34,36,37]. Complimentary information on diastolic dysfunction can be also obtained by passive leg lifting [38,39]. A stiff LV cannot handle such raised filling pressures, hence the resulting pulmonary venous hypertension and symptoms. Similar findings, have been shown by simple leg lifting at rest which results in increased venous return, and a rise in LA pressures corresponding to raised PCWP [38,39].

E/e′ ratio is less age-dependent than mitral E and A waves and an average E/e′ > 14 has a high specificity for increased LV filling pressures. Despite that, many patients have an E/e′ ratio between 8 and 14, due to its low sensitivity, so in this grey zone, other investigations are necessary [40]. E/e′ ratio could be distorted by surgical rings, prosthetic valves, mitral calcifications and regurgitation, and its accuracy is still uncertain in patients with advanced HF and very low CO and large LV volumes [29]. The amelioration of diastolic function as a result of the optimized therapy may also improve the forward flow, i.e., CO and CI, as was recently demonstrated in patients either with acute and chronic HF irrespective of LV EF [41,42].

Although parameters from pulsed wave Doppler mitral, PVF, TDI and estimated pulmonary artery pressures have provided noninvasive means for evaluating LV filling pressures, they have been limited by their accuracy and degree of reproducibility, because of heart rate impact on time relations and the commonly seen arrhythmias and the fact that they cannot be generalized to patients irrespective of LV EF [43,44]. Such limitations highlight the need for using a multiparametric approach, especially in patients with preserved or mildly reduced EF.

To overcome the aforementioned limitations of resting Doppler echocardiography, alternative cardiac ultrasound techniques may be utilized for detecting raised LV filling pressures either in patients with reduced or preserved LV EF. In order to obtain meaningful clinical information, it is important to incorporate diastolic flow data with the clinical findings and possibly with the assessment of circulating natriuretic peptides.

Resting echocardiographic findings may not always succeed in explaining the symptoms/signs of patients presenting to HF clinics, for various reasons, one of which is that symptoms are usually exertional, therefore likely reflecting a different physiological status to that existing at the time of the Doppler echocardiographic examination. For this reason, stress echocardiography has been proposed as the test of choice in assessing patients with exertional symptoms [45]. Stress echocardiography has also been shown to play an important role in patients with a history of HF either to unmask the presence of diastolic dysfunction or for prognostic stratification [46,47]. A number of technical limitations using exercise/stress echo still remain, especially with fast heart rate and breathing disturbances. To avoid such limitations, a moderate exercise test at a heart rate of 100–120 beats per minute could provide the development of disturbances enough to support the diagnosis of raised LV-filling pressures that are responsible for the symptoms.

## 7. Left Atrial Function in Heart Failure

LA function is closely related to LV function since it is one of the main determinants of LV filling, and it contributes to maintain SV and to decrease the filling pressure. The increasing evidence and advances in cardiovascular imaging provide better understanding of LA function, realizing the important role it plays in HF, irrespective of EF (HFrEF and HFpEF) [48,49,50].

LA function is conventionally divided into three phases: reservoir, conduit and pump function.

(1) Reservoir is an expansion phase during LV systole and isovolumic relaxation that permits LA filling from the pulmonary veins. This component is modulated by LA relaxation, LA stiffness and LV longitudinal shortening.
(2) Conduit is the phase in which the blood passively passes from LA to LV. This component is primarily determined by LV diastolic properties (relaxation and compliance).
(3) The pump function consists of the LA actively emptying during late LV systole. Such contractile function is modulated by LV compliance and LVEDP. When the afterload increases, the LA pump function is augmented as a compensatory mechanism to prevent HF. The LA contractile function is also determined by its intrinsic myocardial contractile function, which might decrease when the LA dilates and exceeds the Frank–Starling mechanism.

**Figure 5:**
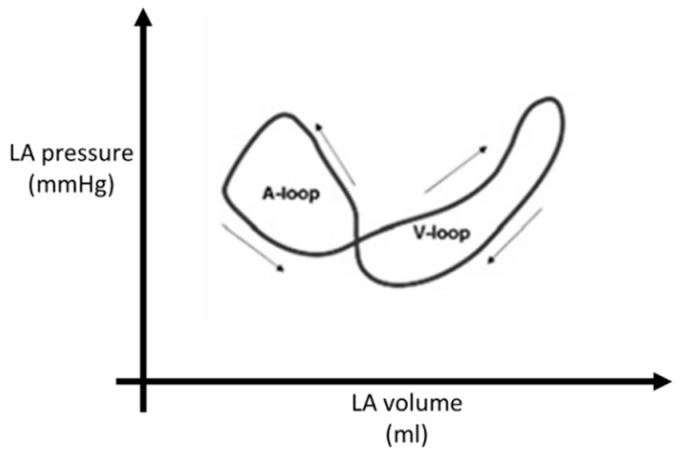
Left atrial pressure–volume relationship consisting in a double loop.

Knowledge of the LA pressure–volume relationship forms the basic understanding of LA mechanical function. The normal LA pressure–volume curve forms a double loop consisting of the A loop (which represents the LA active function) and V loop (which corresponds to passive atrial filling) (Figure 5) [51]. However, invasive measurements are required to evaluate this relationship.

**Figure 6:**
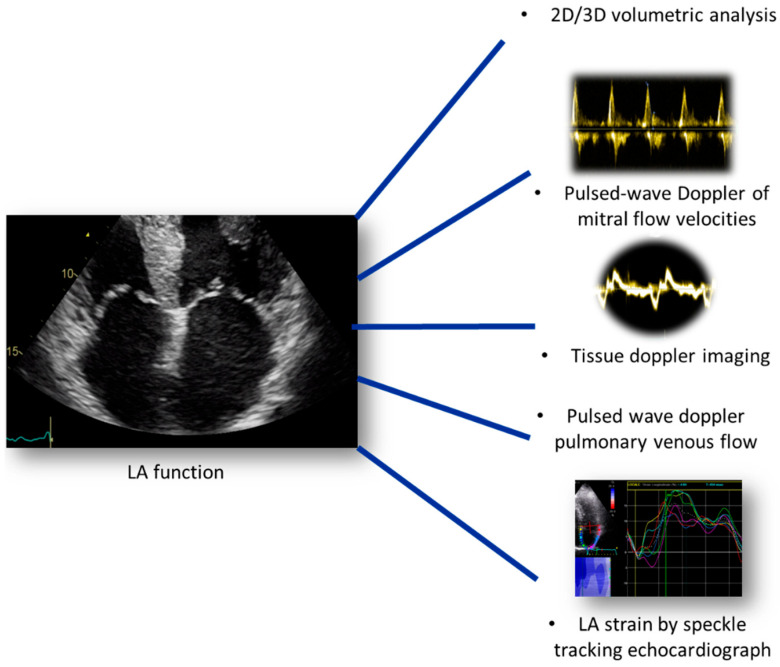
Echocardiographic parameters for the assessment of LA function in a patient with HF.

Echocardiography, in contrast, represents a simple, bedside and non-invasive tool that is useful for the assessment of LA function. Conventional parameters including volumetric analysis, pulsed-wave Doppler of mitral flow velocities, TDI and PVF, associated with STE analysis, are the main indices that allow accurate evaluation of LA pathophysiological changes that associate diastolic dysfunction and HF [52,53] (Figure 6).

## 8. Assessment of Left Atrial Function

Two-dimensional estimation of LA volume is performed at LV end-systole using the biplane disk summation technique. LA volume is indexed to body surface area (LAVi): its upper normal limit by 2D echocardiography is defined as 34 mL/m^2^ [54]. LA volume could also be quantified by 3D transthoracic echocardiography which proved more accurate compared with CMR and has superior prognostic ability [54]. LA dilation represents an independent predictor of mortality, HF and atrial fibrillation. It is also a marker of the chronicity of LV diastolic dysfunction; LAVi is adequate for estimating the chronic effect of elevated filling pressures, but it has low sensitivity in detecting early increases in LV filling pressures [34,54]. Therefore, it should always be considered in combination with other echocardiographic parameters. LV filling velocities by pulsed-wave Doppler, which represent the LA emptying, can be easily used to estimate relative LA function and LV filling pressures. LV E/A ratio estimates the relative contribution of atrial pump function with low E/A ratio (≤0.75) consistent with normal LA pressure and E/A ratio ≥ 2.0 suggestive of high LA pressure [46,50,55].

## 9. Left Atrial Deformation and Raised Filling Pressures

Strain analysis by STE has been used in a variety of cardiac diseases associated with diastolic dysfunction to evaluate myocardial deformation. This ultrasound modality is less dependent on extrinsic variables and therefore more accurate in characterizing intrinsic myocardial properties and it has recently been applied to the quantification of longitudinal myocardial LA deformation dynamics [56].

LA deformation analysis by STE was recently proposed as an alternative approach to estimate LV filling pressures. A close negative correlation between global peak atrial longitudinal strain (PALS) and the LV filling pressure was found. The potential mechanism of this inverse correlation could be explained by the principle that LV filling pressure is the afterload of LA function; if LA pressure is high, the LA should be chronically stressed, resulting in a decrease in LA reservoir function and finally in remodeling with LA chamber dilation, as demonstrated in patients with heart failure [57].

LA strain by STE is a non-Doppler, angle-independent, and objective technique that quantifies LA myocardial function by analyzing myocardial deformation throughout the cardiac cycle. LA strain adds valuable information to conventional echocardiographic parameters, improving its diagnostic accuracy and prognostic stratification value in HF patients [58].

**Figure 7:**
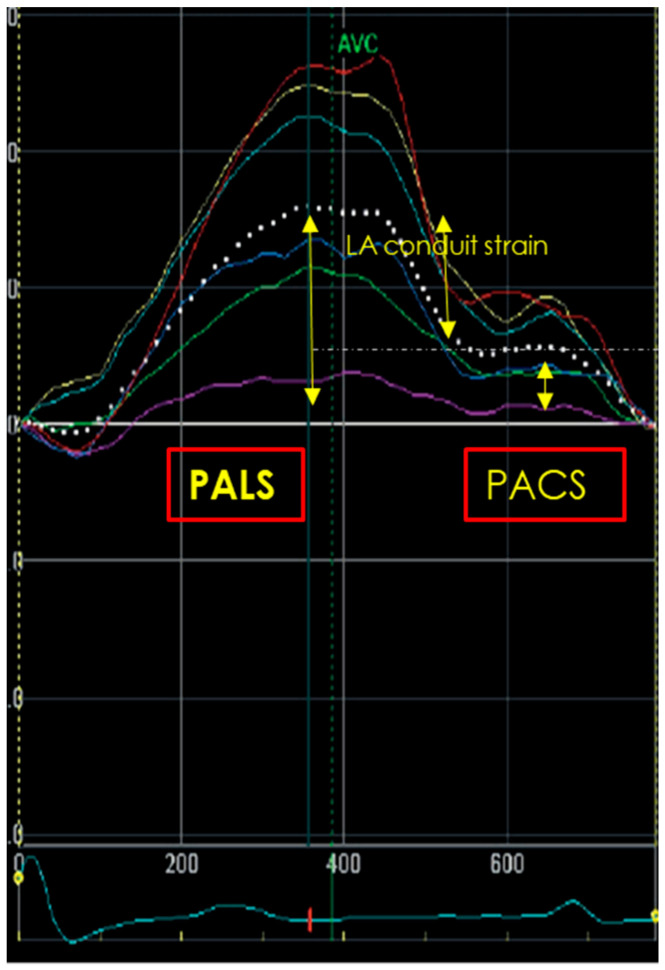
Measurements of peak atrial longitudinal strain (PALS), peak atrial conduit strain and peak atrial contraction strain (PACS).

LA strain assessment requires dedicated LA apical four- and two-chamber view (optional) image acquisitions, avoiding LA foreshortening. A good quality ECG trace and three-consecutive heart cycles are mandatory. P-wave or QRS could be used as the reference starting point. However, based on the results of a multicenter study [59], the European Association of Cardiovascular Imaging/American Society of Echocardiography have recommended the use of QRS onset as the preferred measurement method. Then, dedicated software, that can reduce the need for intervention by the operators, should be used in order to trace the LA endocardium and identify six segments which can be manually adjusted in width and shape. A region of interest (ROI) of 3 mm is recommended, but might require individual adaptation. After optimum tracking, the motion of the ROI should reflect the motion of the underlying tissue. Common problems include failure of the ends of the ROI to follow the mitral annulus, a case that requires adjustments. After final acceptance of the traced ROI, the average curve of all longitudinal strain curves is generated and represents the phases of global LA deformation; in particular, during the reservoir phase, the atrial strain increases, reaching a positive peak at the end of atrial filling. Following mitral valve opening, the LA empties quickly and so the atrial strain decreases, up to a plateau corresponding to the phase of diastasis; then, a second positive peak, less than the first, is generated, corresponding to the period preceding atrial contraction. Three LA strain parameters could be assessed (Figure 7) as follows:

- Peak atrial longitudinal strain (PALS), measured at the end of reservoir phase. This value is always positive and it is about 39% [95% confidence interval CI: 38–41%] in the general population;
- Peak atrial conduit strain, which occurs from mitral valve opening through diastasis until the onset of LA contraction (in patients in sinus rhythm);
- Peak atrial contraction strain (PACS), measured just before the onset of active LA contractile phase. Its reference value is 17% [CI 16–19%] [51,52].

It is well known that LV filling pressure represents the afterload of LA contractile function; hence, when LV filling pressure is raised, the LA could be mechanically stressed and its function compromised. Raised LV filling pressure is reflected in reduced LA reservoir and pump function: many studies have shown that LA reservoir strain is associated with LV filling pressures and an LA reservoir strain < 18% predicts raised LV filling pressures better than LAVi [52]. Moreover, PALS has been shown to have excellent sensitivity and specificity in predicting a PCWP of ≥18 mmHg in patients with HF (PALS < 15.1% had the highest diagnostic accuracy) [60]. Based on this finding, PALS was integrated in the diagnostic algorithm for the evaluation of LV filling pressures by the European Association of Cardiovascular Imaging (EACVI). This algorithm includes mitral E wave associated with E/A ratio and additional criteria when E/A ratio is between 0.8 and 2.0. These criteria include average E/e′ > 14, peak TR velocity > 2.8 m/s and LAVi > 34 mL/m^2^. PALS < 18% is the third suitable parameter when one of the three other criteria is not available and the remaining two are conflicting.

Evaluation of LV filling pressures applying EACVI algorithm can be used in HFrEF as well as HFpEF, despite the fact that the accuracy of LA strain is best in patients with reduced LV EF, and the algorithm cannot be applied in patients with atrial fibrillation [61]. Moreover, this approach for the evaluation of LV filling pressures should be used to obtain a grading of LV diastolic dysfunction according to LV filling velocities and the level of LV filling pressures, crucial for the evaluation of patients with HFpEF (Table 2).

**Figure 8:**
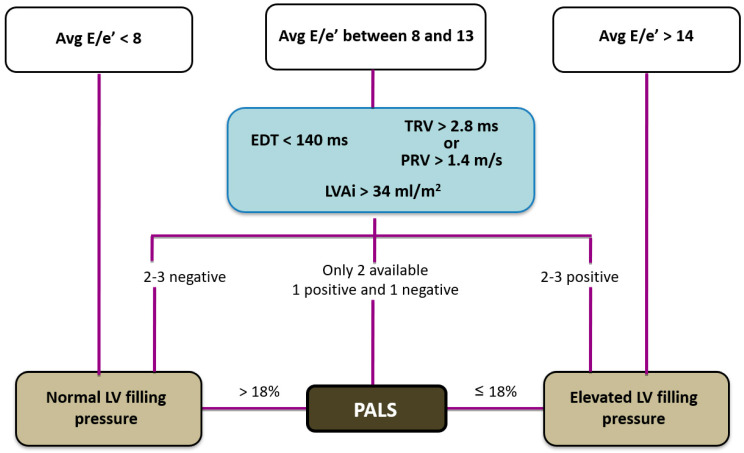
A novel algorithm to estimate elevated LV filling pressure. Legend: Avg E/E′, averaged E/averaged myocardial early velocity; EDT, E wave deceleration time; TRV, tricuspid regurgitation velocity; PRV, pulmonary regurgitation end-diastolic velocity; LAVi, left atrial volume index; PALS, peak atrial longitudinal strain.

As is apparent from the EACVI algorithm, this procedure appears to be too reliant on the assessment of the mitral flow filling patterns, especially on the E/A ratio. In fact, the latter is clearly inadequate in patients with atrial fibrillation, fast heart rate and in those with HFpEF, where an abnormal relaxation pattern can be associated with elevated filling pressures. Therefore, to overcome the limitations of the above mentioned algorithm, a modified system is proposed, that is more focused on the E/e′ ratio and that incorporates parameters like pulmonary regurgitation velocity (Figure 8). Due to the potential of longitudinal myocardial LA deformation analysis, further studies are warranted in any case, to better define its role in this context.

PALS has also been shown to offer additive prognostic information over LV function in acute and chronic HF. Patients with chronic HFrEF and lower PALS had worse event-free survival and developed atrial fibrillation more frequently than those with higher values [62]. Furthermore, PALS has been shown to be a significant prognostic marker in acute HF patients, regardless of HF phenotypes. Other studies have demonstrated that PALS allows accurate prognostication, independent of LA volume and LV longitudinal contraction, a value < 12.9% correlated with an augmented risk of 30% per year adverse event rate [63,64].

In addition, PALS provided important prognostic information in HFpEF: in a cohort of 363 patients hospitalized for dyspnea, the LA reservoir strain discriminated HFpEF from non-cardiac causes of dyspnea, better than all conventional echocardiographic measures. Moreover, worse PALS was associated with reduced CO and decreased peak oxygen consumption (VO_2_), so was able to predict exercise capacity in HF. Finally, PALS showed a strong negative correlation with NYHA class and with NT-proBNP in acute and chronic HF; in fact, the use of PALS as an additive marker of congestion in HF is highly suggested, with the combination of global PALS and NT-proBNP significantly enhancing the prognostic stratification of HF [65,66].

## 10. Right Ventricular Function in Heart Failure

Signs and symptoms of HF could result also from RV dysfunction due to structural and/or functional abnormality: most cases occur as a result of pressure overload (such as in acute pulmonary embolism or in chronic pulmonary hypertension) or volume overload (i.e., tricuspid or pulmonary regurgitations), or decreased RV contractility due to pericardial, myocardial, coronary arteries and/or valve diseases. The RV has a complex 3-dimensional (3D) geometry and its systolic function is complicated, involving not only the longitudinal descent of the tricuspid annulus toward the apex, but also the inward motion of the free wall and apex and of the infundibular tract portion. Echocardiography has become fundamental in the evaluation of RV volume and function with the development of new techniques, such as global longitudinal strain and 3D echocardiography (3DE). Therefore, a composite of multiple parameters is needed to correctly assess the RV. Firstly, the assessment should include RV fractional area change (FAC), which provides an estimate of global RV systolic function (normal values ≥ 35%), the tricuspid annular plane systolic excursion (TAPSE), a measure of RV longitudinal function, and the tricuspid lateral plane systolic velocity by TDI (S′), which is well connected with other measures of global RV systolic function (normal values ≥ 9.5 cm/s) [54]. Moreover, RV longitudinal strain is a useful parameter for the analysis of myocardial deformation and is calculated as the percentage of systolic shortening of the RV free wall from base. RV free wall longitudinal strain (FWLS) has emerged as a good predictor of RV dysfunction after left ventricular assist device (LVAD) implantation and as the most reliable index of RV contractility, knowing the RV physiology (in which the free wall contributes 80% of RV output) and the independence from RV septum/paradoxical septal movement in patients with PH [67]. Adequate RV function is required to ensure antegrade inflow into the device; therefore, RV failure after LVAD implantation remains a significant clinical problem (with rates varying between 5% and 44%, influenced by differing criteria and populations) and burdened with high in-hospital mortality. In a study of 117 patients with end-stage HF, a pre-operative RV free-wall LS < −9.6% predicted post-operative L-VAD RV failure with 76% specificity and 68% sensitivity [68]. Furthermore, RVFWLS also has prognostic value in acute myocardial infarction and pulmonary hypertension [58]. Finally, 3DE has emerged as a good method to quantify RV volumes and EF and as a superior predictor of RV failure compared to conventional parameters in LVAD implantation candidates [67].

## 11. Lung Ultrasound in Heart Failure

Lung ultrasound (LUS) has emerged as a valuable diagnostic and monitoring tool in HF due to its non-invasive nature, portability, and ability to provide real-time information about pulmonary congestion and related findings [69]. LUS has been evolving in recent years, driven by technological advancements, and increasing expertise among clinicians, and has proved to be important. Modern ultrasound machines allow the detailed visualization of lung parenchyma (using low-frequency transducers) and pleural surfaces (using high-frequency transducers). The introduction of point-of-care ultrasound (POCUS) has further revolutionized the use of LUS by enabling rapid targeted assessments at the bedside.

The 2021 ESC guidelines for HF recommends the use of LUS to be integrated with transthoracic echocardiography in the diagnosis and management of HF, especially when NT-Pro BNP is not available, and a new consensus document has been published on the use of LUS in HF [70]. In particular, LUS is highly sensitive for detecting early signs of pulmonary congestion, such as B-lines that are vertical laser-like artifacts, representing thickened subpleural interlobular septa, and are indicative of interstitial syndrome or increased extravascular lung water. Quantifying the number of B-lines per intercostal space provides a semi-quantitative assessment of the severity of congestion, allowing for early intervention to prevent decompensation and hospitalization [71,72,73]. Moreover, LUS findings in HF, such as diffuse bilateral B-lines, often differ from those seen in non-cardiogenic pulmonary edema, such as focal consolidations and pleural effusions. This differentiation is crucial for guiding appropriate management strategies, such as adjusting diuretic therapy or considering alternative diagnoses. Furthermore, serial LUS examinations can track changes in lung congestion over time, providing objective data on treatment response. Reduction in B-lines correlates with clinical improvement and may guide further interventions, while patients with a high number of B-lines at discharge are found to be at a higher risk of readmission or premature death than those discharged without pulmonary congestion or with only mild pulmonary congestion [74,75].

While LUS offers numerous advantages, several challenges and limitations must be considered: firstly, skill and experience in performing and interpreting LUS are essential for accurate diagnosis and monitoring. Secondly, standardized protocols and training programs should help to mitigate operator variability; and its integration with clinical and other imaging data including echocardiography is crucial for comprehensive assessment. Moreover, LUS has limitations in visualizing deeper lung regions and may not detect lesions or abnormalities beyond the pleural surface. Complementary imaging modalities such as computed tomography (CT) may be needed for detailed anatomical assessment in certain cases in the assessment of deep pulmonary consolidation, especially in obese patients.

The role of LUS in HF is continuously evolving, with ongoing research focusing on several areas: the development of automated algorithms for quantifying B-lines and other LUS findings could enhance objectivity and reproducibility, allowing for more standardized assessments across different settings. AI-based systems trained on large datasets have the potential to assist clinicians in image interpretation, risk stratification, and decision-making. Finally, longitudinal studies, including diverse patient populations, will be useful to validate the utility of LUS in guiding HF management, optimizing treatment algorithms, and improving patient outcomes.

## 12. Evaluation of Myocardial Fibrosis

Myocardial fibrosis (MF), characterized by extracellular matrix (ECM) deposition, may result from myocyte loss and/or interstitial proliferation as a result of acute injuries (e.g., myocardial infarction or myocarditis), different chronic pathologies (such as hypertension, valvular heart disease and cardiomyopathy), and it is also present in patients with HFrEF or HFpEF [76,77]. This maladaptive process could explain the initiation of a vicious circle which perpetuates extracellular derangement, myocardial perfusion impairment, and ischemia. MF also contributes to the development of progressive LV diastolic dysfunction [78]. These structural alterations and their pathophysiological counterparts appear to be closely related to the evolution of myocardial failure [79,80,81].

MF could also be a consequence of prolonged and strenuous endurance exercise, notoriously considered to be physiological. It could cause chamber remodeling due to repetitive episodes (during training and competition) of volume overload, myocardial micro-injuries and chronic inflammation, predisposing to atrial/ventricular arrhythmias (most frequently supraventricular tachycardia or atrial fibrillation, AF) [82]. Considering the thinner wall (compared to the LV), the RV and the atria are more susceptible to exercise-induced remodeling (increased volume) and eventually to the development of MF [83].

Regardless of the cause, MF is mostly due to collagen formation and excessive activation of cardiac fibroblasts, which when mild, causes diastolic dysfunction and increasing LV filling pressures and stiffness, but when severe, it may have significant prognostic implications due to severe systolic chamber dysfunction [84]. The process starts with the deposition of the ECM to the detriment of cardiomyocytes inducing excessive activation of cardiac fibroblasts which are responsible for the collagen production and consequently progressive ECM expansion [85,86]. Aortic stenosis (AS) is a typical model of chronic pressure overload resulting in MF; the valve stenosis initially causes LV concentric myocardial hypertrophy as an adaptive response to reduce wall stress but progressively it determines diastolic dysfunction (due to reduced LV compliance and increased chamber stiffness) and reduced LV global longitudinal strain despite a normal LV EF [87]. In severe stages, myocardial contractile function is compromised and deformation impaired, resulting in more severe HF symptoms [88,89]. Hemodynamic adaptations to chronic pressure overload and diastolic dysfunction are also present in the LA with an increase in cavity volume associated with progressive LA longitudinal strain reduction (reduced global PALS), leading to HF symptom development [60]. Aortic valve replacement (surgical or transcatheter) may result in significant recovery of LA structure and function (in the form of reduced indexed LA volume and increased global PALS) consistent with reversed cavity remodeling [90].

In contrast, mitral regurgitation (MR) has a different impact on cardiac function [91]. In the early disease stage, volume overload causes increased myocyte length and stretching, thus increased LV volume overload and eccentric hypertrophy with high volume/low pressure status, followed by progressive LV volume dilation and dysfunction (reduced strain and LV EF). In late stages of the disease, sarcomeric disorganization/disruption occur and finally MF [92]. MR determines LA chronic volume overload and reduced myocardial intrinsic function (reduced global PALS), much earlier than in the LV, due to the thinner LA wall, and also to a greater extent than in the latter, in a similar fashion observed in other conditions such as in diabetes and hypertension [93]. These adverse remodeling events determine myocyte remodeling [94], increased LA stiffness and electrical instability with a high burden of AF which itself contributes to LA remodeling, pulmonary hypertension and HF symptoms. We studied patients with severe MR referred for cardiac surgery, comparing LA myocardial intrinsic function (global PALS) and the extent of LA fibrosis at cardiac biopsy, and we demonstrated a close negative correlation between the two [95]. After mitral valve repair, the LA volume drops, but LA strain showed a residual dysfunction, suggesting that pre-operative LA function does not necessarily reflect the real extent of chamber dysfunction (being masked by volume overload), but rather a process of progressive remodeling and MF [95].

Recent studies have shown that patients with end-stage HF requiring heart transplantation present a high degree of LV and LA MF, as shown by the percentage of MF in histopathological samples (obtained from explanted hearts). In these patients, we demonstrated a significant correlation between the extent of MF and global LV longitudinal strain and to a lesser extent with transverse LV function in the form of circumferential strain and torsion. A cutoff value of GLS of −10.0% had the strongest accuracy for detecting LV fibrosis > 50% with elevated sensitivity and specificity [96]. These findings suggest that MF reflects the drastic changes in myocardial function in end-stage systolic HF, and are able to act as chronic overload on the subendocardium, irrespective of the underlying etiology.

LV MF also results in the loss of synchronous systolic contraction in response to cardiac electrical activation, with healthy myocardial fibers contracting, shortening, and moving toward the cavity center while nonviable and scarred/fibrotic fibers are just tethered or pushed in a dyskinetic fashion, resulting in worse LV mechanical dyssynchrony [97]. Furthermore, the extent of LV fibrosis is independently associated with an unsatisfactory response to medical therapy in patients with idiopathic dilated cardiomyopathy [98]. In end-stage HF patients, MF was present in LA as a result of chronic raised LA pressure, determining myocardial intrinsic dysfunction (reduced global PALS), diastolic dysfunction and contributing to HF symptoms worsening [99].

We have recently compared LA function with the extent of MF on histological samples in patients with end-stage HF and demonstrated that LA fibrosis strongly correlated with global PALS, VO_2_ max, non-invasive LA stiffness, NYHA class with a moderate correlation with invasive LA stiffness and E/e′. Moreover, global PALS proved to be the strongest predictor of invasively assessed severe LA fibrosis (>50%) [99]. This is of clinical importance when explaining the high burden of AF in these patients, suggesting that global PALS could be a future echocardiographic target to optimize medical therapy. In addition, severe LA dysfunction and chronically raised LA pressure correlated with worse symptoms and increased pulmonary pressure, determining RV remodeling and dysfunction with high degree of RV MF; in these patients, RV free wall longitudinal strain was the most accurate measure that correlated with the extent of histological RV MF and functional capacity (VO_2_ max) [100].

## 13. Conclusions

Doppler echocardiographs with all its modalities, conventional and advanced, play an essential role in the management of HF patients, early and late. They accurately estimate filling pressures, predict chamber function recovery and also clinical outcome. An integrated application of all echo modalities should assist in optimizing patient stratification for different procedures, including assistive devices.

## Figures and Tables

**Table 1:** Echocardiographic assessment of systolic and diastolic function in patients with heart failure, detailed on the basis of normal values or ranges and advantages and disadvantages of each method.

		Normal Values or Ranges	Advantages	Disadvantages
Systolic function	LV EF	52–72% in ♂ 54–74% in ♀	✓ Simplicity and good correlation with the patient’s outcome. ✓ Convenient tool in daily clinical practice.	✓ Load dependency. ✓ Lack of any relationship with patients’ signs and symptoms. ✓ Dependency on geometrical assumptions and endocardial border detection.
	LVOT VTI and SVI	LVOT VTI ≥ 15 cm **SVI ≥ 30 mL/m^2^**	✓ A reliable quantitative parameter that truly reflects circulatory status and global end organ perfusion.	✓ Estimation of the real SVI. ✓ Dependence on LVOT diameter assessment.
	LV GLS	LV GLS < −20%	✓ A valuable and more sensitive tool for the evaluation of LV systolic function.	✓ Measurement variability and dependence on image quality and correct acquisition.
	GMW	GCW 1582–2881 mmHg% GWW 226 ± 28 mmHg%	✓ Noninvasive method for evaluating MW.	✓ Dependance on image quality. ✓ Lack of multicenter studies for its validation.
	RV 2D FAC	RV 2D FAC ≥ 35%	✓ Provides an estimate of global RV systolic function, reflecting both longitudinal and radial components.	✓ Fair inter-observer reproducibility.
	TAPSE	TAPSE ≥ 17 mm	✓ Prognostic value.	✓ Partially representative of RV global function.
	TDI S′	S′ ≥ 9.5 cm/s	✓ Highly reproducible. ✓ Good correlation with other measures of global RV systolic function.	✓ Partially representative of RV global function.
	RV FWLS	RV FWLS < −20%	✓ The most reliable index of RV contractility. ✓ Good predictor of RV dysfunction post LVAD implantation.	✓ Dependence on image quality and correct acquisition.
Diastolic function	LAVi	LAVi < 34 mL/m^2^	✓ Adequate for estimating the chronic effect of elevated filling pressures.	✓ Geometric assumptions about LA shape. ✓ Low sensitivity in detecting early increases in LV filling pressures.
	E/A	E/A ≤ 0.75	✓ Easily used to estimate relative LA function and LV filling pressures.	✓ Age-dependent. ✓ Low sensitivity.
	E/e′	E/e′ < 15	✓ The most used non-invasive tool for estimating pulmonary artery pressure	✓ Low sensitivity. ✓ Distorted by surgical rings, prosthetic valves, mitral calcifications and regurgitation.
	PALS	PALS 38–41%	✓ It predicts raised LV filling pressures better than LAVi. ✓ PALS < 15% has good sensitivity and specificity in predicting a wedge pressure ≥ 18 mmHg	✓ Dependence on image quality, rhythm and correct acquisition.

E/A, ratio between mitral E and A wave velocity; E/e′, ratio between E wave and averaged myocardial early velocity on tissue Doppler imaging; LAVi, left atrial volume index; LV, left ventricle; LVAD, left ventricular assist device; LV EF, LV ejection fraction; LVOT VTI, left ventricular outflow tract velocity time integral; GCM, global constructive work; GLS global longitudinal strain; GMW, global myocardial work; GWW, global wasted work; PALS, peak atrial longitudinal strain; RV, right ventricle; RV 2D FAC, RV 2 dimensional fractional area change; RV FWLS, RV free wall longitudinal strain; SVI, stroke volume index; TAPSE, tricuspid annular plane systolic excursion; TDI S′, tissue doppler imaging S wave.

**Table 2:** Grading of LV diastolic dysfunction as proposed by the 2022 EACVI expert consensus document.

	Grade I	Grade II	Grade III
**LV filling pressure**	Low or normal	Elevated	Elevated
Mitral E/A ratio	≤0.8	>0.8 to <2	≥2
